# Anti-leucine-rich glioma-inactivated 1 encephalitis: two case reports and a review of the literature

**DOI:** 10.1186/s13256-022-03650-x

**Published:** 2022-11-09

**Authors:** Sanaz Ahmadi Karvigh, Saeideh Salehizadeh, Fahimeh Vahabizad

**Affiliations:** Department of Neurology, Sina Hospital, Tehran University of Medical Sciences, Tehran, Iran

**Keywords:** LGI1, Faciobrachial dystonic seizure (FBDS), Basal ganglia, Immunotherapy, Rituximab, Autoimmune encephalitis

## Abstract

**Background:**

Anti-leucine-rich glioma-inactivated 1 encephalitis is a newly emerged entity characterized by frequent faciobrachial dystonic seizures and a wide spectrum of subacute clinical symptoms such as other seizure types, mood and behavioral changes, and memory loss. We should be aware of differentiating this diagnosis from psychogenic nonepileptic seizures. Mesial temporal, limbic structures, and basal ganglia are the most commonly involved regions.

**Case presentation:**

Here we review the available data, and report on two young Iranian (White) females, 24 and 18 years old, who represent distinct aspects of the disease. The clinical presentation and degree of tissue involvement varies to some extent in the two reported cases. Case 1 had prominent neuropsychiatric symptoms and suffered from frequent faciobrachial dystonic seizures with more significant basal ganglia involvement, whereas case 2 suffered from severe memory decline and dialeptic seizures along with mesial temporal involvement. Symptoms were refractory to usual treatment and prompt immunotherapy was needed.

**Conclusions:**

This disease has a rather favorable outcome provided that treatment is initiated early. However, resistance to first-line treatment, relapses, and long-term complications highlight the need to establish reliable biomarkers to distinguish different subtypes of this disorder to predict the clinical outcome and prognosis, and to refine management.

## Introduction

Autoimmune limbic encephalitis can be associated with different neural-specific autoantigens, of which autoantibodies against the voltage-gated potassium channels (VGKC) are the most common [1, 2]. These can target any of the three subunits of the VGKC complex including leucine-rich glioma-inactivated 1 (LGI1), contactin-associated protein-like 2 (CASPR2), and contactin-2. Encephalitis related to anti-LGI-1 antibody is more common than these three types, and was first described in 2010 [2, 3]. Although it can be a paraneoplastic phenomenon, anti-LGI-1 encephalitis is mostly unrelated to tumors [4]. It mostly affects males in their 60s [5, 6], and can manifest as subacute memory decline, cognitive impairment, psychiatric disturbances, refractory seizures, hyponatremia, and, in some cases, dysautonomia and sleep disturbances [1, 2, 6]. The most common presenting symptoms are seizures (53%) or cognitive disorders (42%). Faciobrachial dystonic seizures (FBDS), if present, are the characteristic seizure type for this disease [3, 4]. The diagnosis is primarily made on the basis of the clinical presentation (mainly subacute refractory seizures otherwise unexplained) and evidence of neurally directed inflammation. The diagnosis is confirmed by finding the anti-LGI-1 antibody and after excluding other possible causes of encephalitis. All the same, underdiagnosis is not uncommon, owing to ambiguous clinical presentation or absence of distinguishing findings. Many of these patients are initially diagnosed with nonorganic psychogenic disorders until the first undeniable generalized tonic–clonic seizure happens [7]. Therefore, several diagnostic tools have been developed in recent years, such as the Antibody Prevalence in Epilepsy and Encephalopathy (APE2) and Response to Immunotherapy in Epilepsy and Encephalopathy (RITE2) scores to help facilitate the detection of the disease and consequently accelerate treatment [8]. Neuroimaging findings are usually more illuminating in the acute phase, whereas changes in cerebrospinal fluid (CSF) analysis are less frequent [6, 9]. In the end, detection of anti-LGI1 antibodies in serum or CSF is needed to confirm the diagnosis [2]. Seizures are commonly refractory to usual antiseizure medications. But, fortunately, they have been eliminated dramatically by immunotherapy, especially steroids. This feature also helps the physician to diagnose the disease [4, 9].

Here, we aim to introduce two patients with different clinical manifestations and distinct localization of brain involvement. Through these two interesting cases, we can split the diagnostic process into four steps: recognizing the spectrum of the clinical presentation, finding the evidence of neurally directed inflammation, excluding other differential diagnosis, and how to proceed with treatment.

## Case presentations

### Case 1

**Fig. 1:**
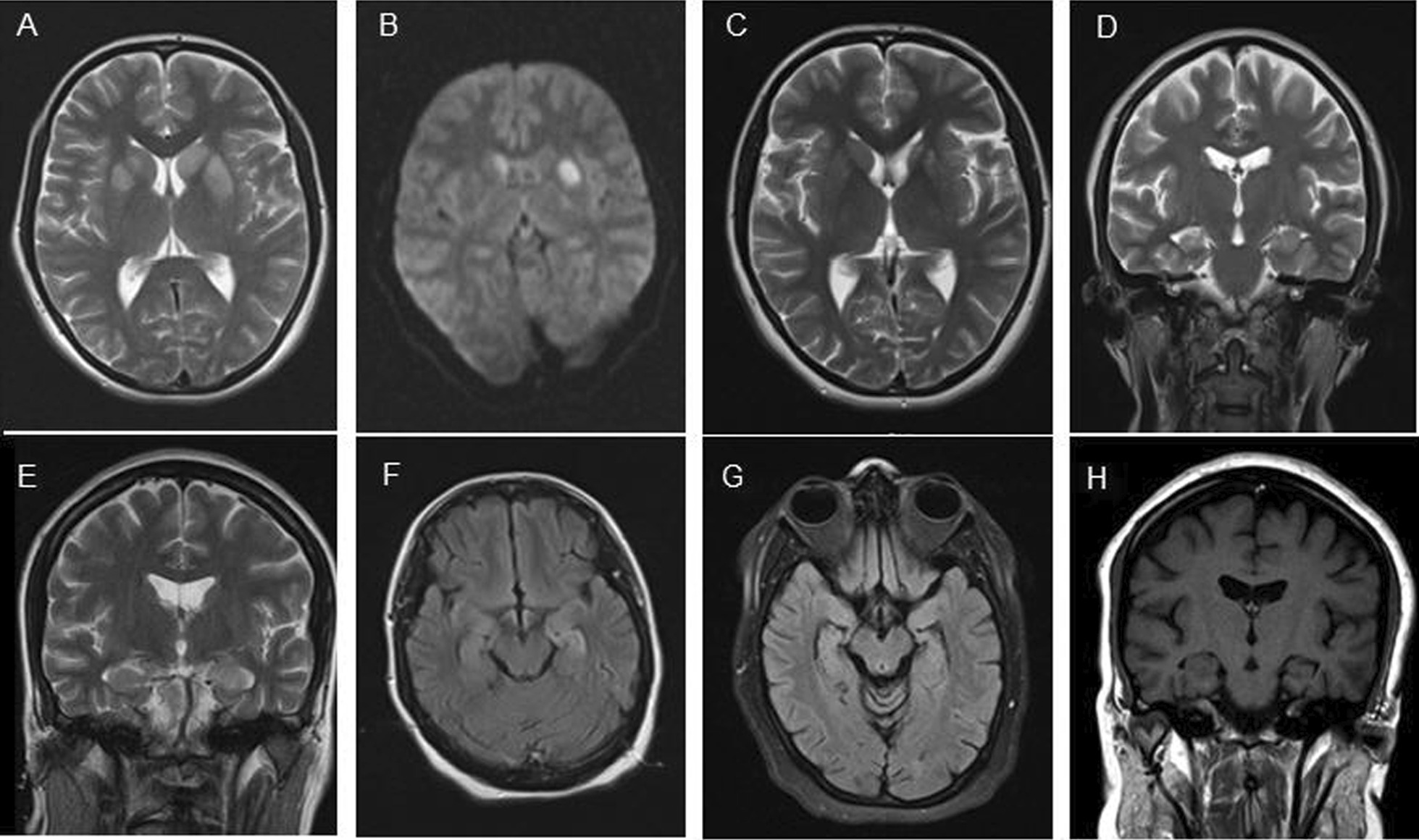
Case 1: bilateral hyperintense lesions in basal ganglia (left more than right) in T2 sequence (**A**). Left basal ganglia restriction in diffusion-weighted imaging (**B**). Significant decrease in Magnetic resonance imaging hyperintensities in the basal ganglia on 6-month follow up Magnetic resonance imaging (**C**) and right hippocampal sclerosis (**D**). Case 2: Transvere relaxation time 2- Fluid-attenuated inversion recovery hyperintensities in the left mesiotemporal structures (**E**, **F**). Mild left-side hippocampal sclerosis on the 1-year follow-up Magnetic resonance imaging (**G**, **H**)

The patient was a 24-year-old Iranian (White) female with a 1-month history of progressive memory decline and behavioral changes including anxiety, visual hallucinations, aggression, and obsessive thoughts. She also reported episodes of mild insomnia and autonomic dysfunctions (presyncope and syncope attacks), which were unusual for her. The day before her admission, she started having episodes of right upper limb jerks that sometimes evolved into sudden turning of the head, spasm of the jaw muscles, and dystonic posturing of her right hand and right lower face without any impairment of awareness, which lasted a few seconds (FBDS and left arm myoclonus). Later on, the right arm was also involved. These episodes occurred 20–50 times per day and continued during sleep. Finally, she had a generalized tonic–clonic seizure on her first day of admission, which she claimed followed one of her prolonged FBDS. There was no history of substance abuse, past comorbidities, or recent drug administration. We could not detect any fever or other abnormalities on systemic examination. The neurologic examination was unremarkable except for a mild recent memory dysfunction. The routine laboratory studies were normal except for a mild decrease in serum sodium level (130 mg/dL). Brain magnetic resonance imaging (MRI) showed hyperintense lesions in the bilateral (left more than right) basal ganglia (BG) in T2 and fluid attenuated inversion recovery (FLAIR) sequence without enhancement after gadolinium injection. There was evidence of left BG restriction in apparent diffusion coefficient-diffusion-weighted imaging (ADC-DWI) MRI. Similarly, both hippocampi showed minor degrees of involvement (Fig. 1A, B).

Serologic investigations showed no evidence of drug toxicity, inflammation, or infection. CSF analysis revealed a normal opening pressure, with normal glucose and protein, and five leukocytes (all lymphocytes).

Oligo-clonal bands (OCB) were detected in the CSF. CSF screening for herpes simplex virus 1 and 2, *Cryptococcus* and *Mycobacterium tuberculosis* was negative. However, anti LGI-1 antibody was detected both in the serum and CSF. Malignancy workup was negative. The scalp electroencephalogram (EEG) showed moderate diffuse encephalopathy with frequent bursts of frontal intermittent rhythmic delta activity (FIRDA) activity without any epileptiform discharges. On continuous video-EEG monitoring, the FBDS correlated with no clear EEG ictal pattern. Treatment with antiseizure medications (sodium valproate, levetiracetam, phenytoin) successfully controlled the seizures. We avoided using carbamazepine, which could aggravate the existing hyponatremia. Nevertheless, the frequent FBDS did not respond to treatment, and increased even after the steroid pulse therapy (1 g/day methyl prednisolone intravenously for 5 days followed by 60 mg/day oral prednisolone). Therefore, despite marked cognitive and mental improvement with steroids, we escalated the treatment to a full course of intravenous immunoglobulin (IG; 2 g/kg for 5 days). She was then discharged with oral prednisolone 50 mg/day, valproate 1600 mg/day, levetiracetam 2500 mg/day, clobazam 30 mg/day, and prophylactic co-trimoxazole. Three weeks after this treatment, although the frequency of seizures decreased significantly, she still had more than 10–20 FBDS per day that mostly disrupted her night sleep. Adding lacosamide did not have a clear effect on seizure control. Therefore, rituximab was introduced (1000 mg intravenously once every 2 weeks for two doses). The oral prednisolone level was gradually tapered to 10 mg/day. After 3 weeks, she reported complete cessation of the FBDS, and there were no residual cognitive or behavioral symptoms. Her CD19 and CD20 levels were < 0.1. There was no adverse effect after rituximab administration except for a transient severe leukopenia total neutrophil count of 132/mm^3^) in the first month, which was not complicated by clinical infection and was treated with prophylactic empiric antibiotics and granulocytic colony stimulating factor (G-CSF).

There was no relapse, and antiseizure medications were tapered gradually within the first year and discontinued later on. The oral prednisolone 5 mg every other day was discontinued after 9 months. The last LGI-1 serum Ab was undetectable and CD19-20 levels remained < 0.1 for 12 months. Therefore, we did not repeat the rituximab injection. Follow-up EEGs were normal. Although the MRI hyperintensities in the basal ganglia were markedly decreased, there was evidence of right hippocampal sclerosis (HS) on 6-month follow-up brain MRI (Fig. 1C, D). The psychiatric and cognitive disturbances resolved completely after 6 months of immunotherapy, and the antidepressants and antipsychotic medications were discontinued. The patient has been followed-up for 2 years. After 1 year of complete withdrawal of antiseizure medication and immunomodulatory treatment, so far there has not been a flare up in the disease, an increase in anti-LGI-1 serum level, or seizure recurrence.

### Case 2

**Fig. 2:**
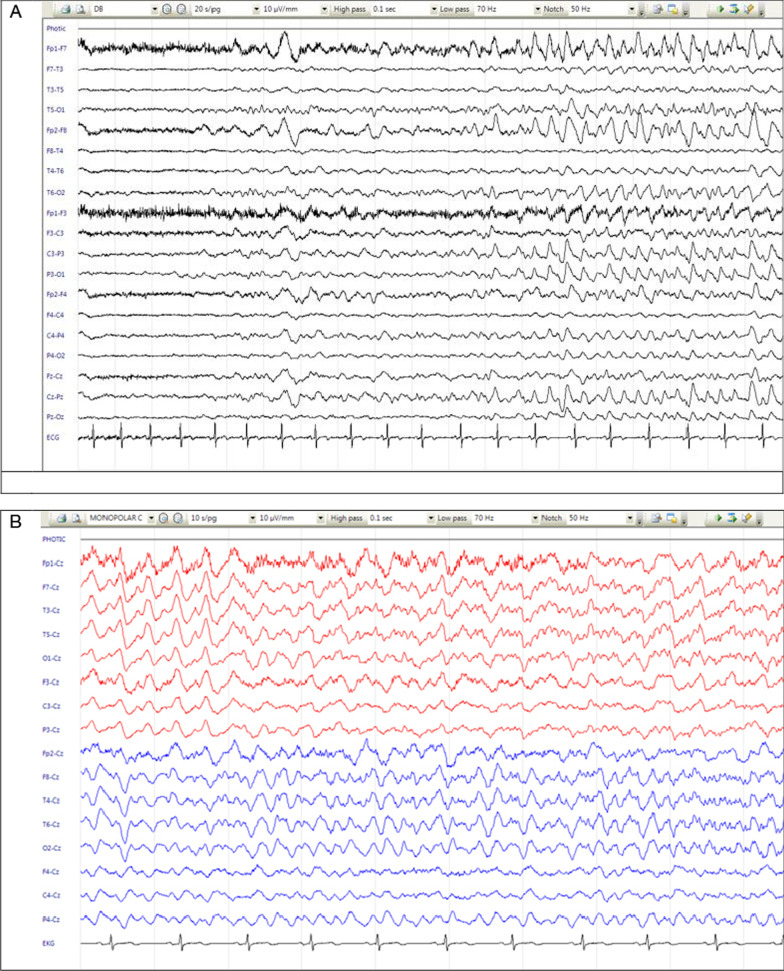
Case 2: nonconvulsive status epilepticus: electroencephalogram ictal pattern starting from FP1, F7 (**A**). Focal electroencephalogram ictal pattern independent in the left (F7) and right (F8) temporal region (**B**)

Our second patient was an 18-year-old Iranian (White) female who presented with frequent episodes of brief amnesia, unresponsiveness, and activity arrest (focal cognitive seizures with impaired consciousness or dialeptic seizure with loss of awareness) for about 1 week prior to admission. She also suffered from profound memory impairment, mild insomnia, and severe dysautonomia (presyncope and syncope episodes). Since the seizures did not have motor manifestations, they were neglected by the family and signed off as psychiatric disturbances, until she had an episode of generalized tonic–clonic seizure (GTCS) and was brought to the hospital. On the second day of admission, the focal cognitive seizures became really prolonged, which was compatible with a nonconvulsive status epilepticus proper (focal cognitive/dialeptic status with impaired awareness, nonconvulsive status epilepticus). The EEG showed a repetitive multifocal EEG ictal pattern lasting 10–40 seconds with origin from Cz, Pz, or F8, T4 or F7, and T3 occurring every 40 seconds. Between the seizures, the background was slow and consisted of delta–theta activity (Fig. 2). The nonconvulsive status epilepticus (NCSE) was successfully controlled with intravenous phenytoin and levetiracetam. However, occasional seizures still occurred, which prompted us to add sodium valproate to the antiseizure treatment. Meanwhile, routine serologic investigations ruled out underlying toxic metabolic abnormalities. The brain MRI showed significant T2-FLAIR hyperintensities in the left mesiotemporal structures without gadolinium enhancement or DWI restriction (Fig. 1E, F).

The CSF study showed normal intracerebral pressure (ICP) with 10 lymphocytes, elevated protein level (64 mg/dL), normal glucose level, and positive OCB. The CSF infectious panel was negative, similar to case 1. The anti-LGI-1 antibody was detected in the serum and CSF, and the diagnosis was confirmed. Treatment was initiated with corticosteroid pulse therapy, followed by intravenous IG. There was a slight improvement in the clinical features. Three weeks after intravenous IG treatment, owing to incomplete resolution of seizures, rituximab was administered. After 2 weeks, there was complete remission of seizures. The cognitive and psychiatric problems were almost resolved. Maintenance treatment with valproate, levetiracetam, and lamotrigine was continued for 6 months and then gradually withdrawn. We continued the oral prednisolone (25 mg every other day) for 6 months before tapering it off. The CD19–20 count started to drop after 1 month and remained less than 0.1 for 14 months. In this period, there was no relapse, therefore no repeat dose of rituximab was needed. There was a complete resolution of cognitive and psychiatric symptoms. The patient has been off all medication for 12 months. So far, there has not been a flare up in the disease, increase in anti-LGI-1 serum level, or seizure recurrence. However, there is a mild left-side hippocampal sclerosis on the 1-year follow-up brain MRI (Fig. 1G, H). Since there were no clinical seizures or epileptiform abnormality in the EEG, we decided not to treat her with any antiseizure medication.

## Discussion

### Step 1: clinical presentation

LGI1-encephalitis mostly occurs in middle-age and older adults. In fact, the median age of onset is around 63 years of age. The male-to-female ratio of LGI1-encephalitis is 2:1 [6, 10]. Conversely, our patients were young females. There is a wide spectrum of clinical symptoms in LGI1-encephalitis, ranging from seizures (FBDS, GTCS), subacute mood and behavioral changes, cognitive disorders, and memory loss [3]. This can be affected by the location of CNS involvement. Although diencephalon, insular cortex, and other scattered neocortical regions can be involved, the mesial temporal limbic structures and basal ganglia are the two important regions affected in anti-LGI1 encephalitis, and they are associated with distinctive symptoms [11, 12]. As expected, focal cognitive seizures and memory disturbance are attributed to lesions of the hippocampus, whereas basal ganglia lesions can cause psychiatric (delusions and paranoia) and abnormal movement symptoms. The peculiar nature of the FBDS seems to be a result of epileptic activity in the network connecting the basal ganglia to the motor cortex [13, 14]. It has been shown that, during the ictal phase of these seizures, the EEG may be normal or show slow waves in the bilateral or contralateral frontal leads [7, 15, 16]. Although the absence of marked epileptic activity in EEG during FBDS has led to mislabeling it as a psychologic event, in fact, the normal ictal scalp EEG supports the assumption of a deep anatomic origin of the FBDS [7]. In the first case, there was no evidence of epileptic discharges. Nevertheless, cortical involvement can sometimes give rise to EEG abnormality in favor of epilepsy, as seen in the second case. In case 1, who had prominent neuropsychiatric symptoms and suffered from frequent FBDS, it was seen that the basal ganglia involvement was more significant compared with the mesial temporal involvement seen in case 2, who had severe memory decline and dialeptic seizures.

Vigilance is required by the physician to suspect this diagnosis when encountering the aforementioned set of clinical symptoms with a subacute refractory course, as well as otherwise unexplained underlying etiologies.

### Step 2: confirming the diagnosis

The paraclinical findings can indicate the presence of an inflammatory process, which can be nonspecific or neural specific. Needless to say, extensive serologic investigation helps exclude other differential diagnoses.

As shown in case 1, hyponatremia is often present in anti-LGI-1 encephalitis [1, 2]. Brain MRI findings may vary from patient to patient, and the hippocampal involvement, either bi- or unilateral, would be the most expected finding [12]. High T2/FLAIR signal in the mesiotemporal lobes may be characteristic. However, normal MRI is not uncommon. The lesions have the characteristics of an inflammatory lesion, showing hyperintensity in T2 and FLAIR with occasional gadolinium enhancement and swelling [3, 12, 13]. The restriction in DWI sequences, which is suggestive of cytotoxic edema, is only noted in very severe cases (such as case 1), but does not always mean a permanent sequela. Swelling and hypersignal changes in the medial temporal lobes in the T2 sequence, especially in the acute phase, were reported in 50–74% of patients [11, 13, 17]. One study initially revealed a hippocampal hyperintensity in 42% of patients, and in 70% of patients at later stages [12]. Both of our patients revealed hippocampus involvement at the initial stages. They also developed HS later on. The MRI abnormalities accompanying FBDS are not well described, but sometimes signal changes are evident in unilateral or bilateral basal ganglia, as seen in case 1. In one study, there was evidence of BG involvement in 28% of patients [7, 13]. Hence, MRI can be a useful tool to confirm the basal ganglia localization of brain involvement in anti-LGI-1 encephalitis patients with FBDS [7]. The CSF analysis can also provide evidence of inflammation, such as a slight increase in the white blood cells (WBCs), which is mostly lymphocytic with a mild increase in the protein level [6, 9]. Intrathecal OCB and increased IgG index were detected in a minority of patients when compared with other autoimmune encephalitis. The intrathecal synthesis of antibodies is not prominent here. The final step is to detect the anti-LGI-1 antibody. Presence of anti-LGI-1 antibodies in the serum appears to be more likely than presence of the same in the CSF [3, 10, 18]. The current scarce available data show a significant correlation between the clinical disease activity (relapses) and antibody levels [3, 10, 18]. It is important to note that the antibody titer is not related to the long-term cognitive outcome [19].

### Step 3: excluding other differential diagnosis

While performing the above evaluations, most of the other etiologies can be excluded. The most important ones to investigate, especially in the absence of the characteristic FBDS, are infectious encephalitis [such as herpes simplex virus (HSV)], toxic metabolic derangement, tumors, vascular insults, and so on.

### Step 4: treatment and prognosis

Symptoms, especially seizures, are refractory to usual treatments, and immunotherapy should be used for their alleviation. Although the response to immunotherapy is dramatic and can be used as a confirmation for the diagnosis, most patients with limbic encephalitis would need several weeks to fully respond to therapy. Often escalation to the next line of treatment is warranted. The first line of treatment is usually corticosteroids in 89.5% of cases, followed by intravenous IG and then plasmapheresis [4, 19, 20]. The second line of treatment (including rituximab, cyclophosphamide, mycophenolate mofetil, azathioprine, and tacrolimus) is used in a limited proportion of patients with anti-LGI-1 encephalitis. Anti-LGI-1 encephalitis usually responds well to the first line of treatment with steroids [4]. About 78% of patients that are treated with intravenous immunoglobulin, plasma exchange, corticosteroid, rituximab, azathioprine, and cyclosporine have a good clinical outcome. Full recovery or mild residual memory impairment followed by full return to work is expected. Recurrence is not common for this disease process. Nonetheless, relapses have been reported and careful follow-up and cautious tapering of immunotherapy is recommended [9, 21, 22]. We noted that complete resolution of symptoms was achieved only after administration of rituximab. It is important to note that LGI-1 is a surface synaptic neural antigen, and B-cell-mediated humoral immunity plays a significant role in the disease pathogenesis [6, 21]. On the other hand, rituximab, which is a monoclonal antibody that binds to the CD20 antigen on B lymphocytes, can effectively moderate the B-cell immunity with an acceptable safety and tolerability profile [23–25]. Therefore, we can infer that rituximab may have a promising place in the treatment of anti-LGI-1 encephalitis. We also noted that in both of our patients, the anti-LGI-1 antibody was strongly present in the CSF with positive OCB, although we could not measure the exact antibody level and, by extension, the intrathecal synthesis index either. This finding, which may be due to marked intrathecal antibody synthesis in our patients, led us to a hypothesis that the presence of antibodies and OCB in the CSF may be used as an indicator of disease severity or response to treatment, which can be examined in future research. However, an unusual complication of rituximab therapy, rituximab-associated neutropenia (RAN), has been described recently. It may take place either during treatment or several months after it. Circulating antibodies in the plasma may be responsible for this unique bone marrow toxicity [23]. In case 1, we managed this asymptomatic side effect with G-CSF and prophylactic antibiotics without any further adverse effects.

Although epilepsy after resolved encephalitis is rare in patients treated with immunotherapy, symptomatic management of seizures with antiseizure medication should also be kept in mind. Various medications have been recommended, including levetiracetam, sodium valproate, phenytoin, carbamazepine, and so on. Sodium channel blockers such as carbamazepine have been more promising in controlling seizures [26]; however, hyponatremia should be meticulously monitored in these patients [27]. There have been some doubts regarding the use of levetiracetam in these patients owing to unoptimized seizure control and an adverse effect profile that could mimic a flare-up [26, 28]. On the contrary, our patients did not report any complications when using levetiracetam as an antiseizure medication.

## Conclusion

Although long-term follow-up of LGI1 encephalitis without immunotherapy has not yet been investigated comprehensively, about 78% of patients who received these treatments have a good clinical outcome with full recovery in 1 year, and may have mild residual memory impairment [19]. It has been well documented that rapid initiation of treatment in autoimmune encephalitis corresponds to better short- and long-term outcomes and helps prevent irreversible lesions, such as hippocampal atrophy [12]. We believe that in cases 1 and 2, who had a rather severe disease, the early administration of rituximab proved to invoke a dramatic response, both in eliminating the acute phase symptoms and in preventing long-term complications and relapses, without any significant side effects. Therefore, we strongly recommend early administration of rituximab in the management of anti-LGI-1 encephalitis, although more controlled trials are needed to corroborate this recommendation. The anti-LGI-1 encephalitis can present with different clinicopathological features that differ in disease severity and treatment response. It is also important to find a reliable biomarker to distinguish a specific group of patients who may benefit from a tailored treatment with rituximab.

## Data Availability

The datasets used and/or analyzed during the current study are available from the corresponding author on reasonable request.
